# Commentary: Honoring our teachings: children's storybooks as indigenous public health practice

**DOI:** 10.3389/fpubh.2025.1689247

**Published:** 2025-10-30

**Authors:** Yulu Jiang, Hao Gu

**Affiliations:** School of Fine Arts and Design, Yangzhou University, Yangzhou, China

**Keywords:** culturally grounded, American Indian/Alaska Native, COVID-19, storytelling, indigenous research

## 1 Introduction

Maudrie et al. employed the example of “Our Smallest Warriors, Our Strongest Medicine: Honoring Our Teachings during COVD-19,” which argued for the legitimacy and effectiveness of embedding Indigenous storytelling traditions into public health communication. The research team collaborated with multiple tribal entities to distribute over 50,000 printed books and online resources at no cost, supplemented by 34 caregiver questionnaires. These questionnaires revealed remarkably high satisfaction and knowledge acquisition rates. Four “storybook” medium, responses to internal differences within Indigenous communities, and long-term health impact assessments, all of which warrant further exploration.

## 2 Subsections

### 2.1 The double-edged effect of storybooks as a medium

Storybooks serve a dual function of providing emotional security and knowledge transfer in shared reading situations between children and parents, but the author does not adequately discuss the potential barriers to reading posed by literacy rates and language diversity. The oral traditions of American Indian and Alaska Native (AI/AN) communities remain strong, with some remote tribes having English literacy rates below the national average, and 31% of households lacking reliable internet access. If storybooks are used as the primary vehicle for public health initiatives, this could further exacerbate the information gap. Future efforts could consider developing audio versions, tribal language podcasts, and community theater performances to address the limitations of text through “multimodal storytelling.”

### 2.2 The “pan-indigenous” paradigm and the variances among tribal entities

The article emphasizes “cross-tribal shared values,” but it is difficult to avoid the risk of “indigenization.” The 574 federally recognized tribes differ significantly in language, governance, beliefs, and experiences during the pandemic. For example, the collective ritual taboos of the Southwest Pueblo tribes and the winter gift-giving rituals of the Northwest Coast tribes are not consistent with pandemic prevention logic. While the storybook features diverse characters, its plot primarily follows a “family-community-ancestors” triadic structure, which may obscure the unique circumstances of urban Indigenous peoples or tribes without traditional territories. Future public health storytelling initiatives should establish a “tribal self-determination review” mechanism, where older population or youth councils from the target community review each page for cultural symbols and language use to ensure “cultural safety” rather than one-way dissemination.

### 2.3 A comprehensive evaluation of the design and its long-term health implications is imperative

The current assessment relies on voluntary, convenience sampling (*n* = 34) and does not track changes in children's mental health indicators over time. The potential alleviating effects of storybooks on anxiety, depression, or post-traumatic symptoms remain speculative. A mixed-methods approach is recommended: (1) Quantitative: Collaborate with tribal schools to measure children's emotional symptoms using scales such as the RCADS and CDI-2 3 months before and after distributing storybooks; (2) Qualitative: Conduct focus groups to explore how children translate the “Fourfold Teachings” from the books into daily coping strategies.

Additionally, attention should be given to the “story saturation” phenomenon—where the marginal utility of information diminishes after repeated readings. This can be addressed through “story continuation” workshops where children collaborate with older population to co-create subsequent chapters, thereby facilitating knowledge reproduction. For additional requirements for specific article types and further information please refer to “Article types” on every Frontiers journal page.

## 3 Discussion

Maudrie et al.'s work demonstrates the immense potential of storybooks as a rapid, low-cost, culturally appropriate public health instrument during times of crisis. However, the transition of this model from a “pandemic response” to a “routine health promotion” strategy necessitates the consideration of three fundamental challenges: literacy disparities, tribal diversity, and assessment depth. It is imperative for public health researchers to establish long-term collaborations with tribal colleges, language revitalization projects, and digital equity initiatives to collectively develop a “decolonized” knowledge dissemination ecosystem. In this ecosystem, stories are not merely read but also narrated, adapted, and reimagined, thereby genuinely “honoring our teachings.”

The mixed-methods evaluation we advocate must be grounded in Indigenous research methodologies and co-designed and interpreted with full community participation to avoid exploitation and ensure cultural appropriateness. This stance embodies reciprocity, respect, and relevance—indispensable cornerstones for any public health intervention within the IRM paradigm.

